# Up-regulation of KIF14 is a predictor of poor survival and a novel prognostic biomarker of chemoresistance to paclitaxel treatment in cervical cancer

**DOI:** 10.1042/BSR20150314

**Published:** 2016-04-05

**Authors:** Wenjing Wang, Yanhua Shi, Jing Li, Wei Cui, Baozhi Yang

**Affiliations:** * Jinan Second People's Hospital, No. 148 Jingyi Road, Jinan 250011, Shandong, China; † The Second Hospital Affiliated to Shandong University of Traditional Chinese Medicine, No. 1 Jingba Road, Jinan 250001, Shandong, China

**Keywords:** cervical cancer, chemoresistance, KIF14, prognosis

## Abstract

Kinesin family member 14 (KIF14) is a member of kinesin family proteins which have been found to be dysregulated in various cancer types. However, the expression of KIF14 and its potential prognostic significance have not been investigated in cervical cancer. Real-time PCR was performed to assess the expression levels of KIF14 in 47 pairs of cervical cancer tissues and their matched normal tissues from patients who had not been exposed to chemotherapy as well as tissue samples from 57 cervical cancer patients who are sensitive to paclitaxel treatment and 53 patients who are resistant. The association between KIF14 expression levels in tissue and clinicopathological features or chemosensitivity was examined. Kaplan–Meier analysis and Cox proportional hazards model were applied to assess the correlation between KIF14 expression levels and overall survival (OS) of cervical cancer patients. KIF14 expression levels were significantly increased in cervical cancer tissues compared with matched non-cancerous tissues and it was higher in tissues of patients who are chemoresistant compared with those who are chemosensitive. KIF14 expression was positively associated with high tumour stage (*P*=0.0044), lymph node metastasis (*P*=0.0034) and chemoresistance (*P*<0.0001). Kaplan–Meier analysis showed that high KIF14 expression levels predicted poor survival in patients with (*P*=0.0024) or without (*P*=0.0028) paclitaxel treatment. Multivariate analysis revealed that KIF14 was an independent prognostic factor for OS. Our study suggests that KIF14 may serve as a predictor of poor survival and a novel prognostic biomarker of chemoresistance to paclitaxel treatment in cervical cancer.

## Abstract (precis)

KIF14 may serve as a predictor of poor survival and a novel prognostic biomarker of chemoresistance to paclitaxel treatment in cervical cancer.

## INTRODUCTION

Cervical cancer is the second most common gynaecological malignancy after breast cancer in the world and is the major cause of death from gynaecological cancers in developing countries like India [1–3]. It is estimated that approximately 12900 new cases of invasive cervical cancer will be diagnosed and 4100 women will die from cervical cancer in the year 2015. It develops in a stepwise manner and involves a sequential progression from normal cervical epithelium to preneoplastic cervical intraepithelial neoplasia and then to invasive cervical cancer [4]. Although increasing evidence suggests that early detection by testing for high-risk human papillomavirus (HPV) and cervical papilloma smears have reduced cervical cancer mortality, these methods do not monitor the development of cervical cancer directly [5]. Therefore, identification of novel biomarkers is needed to improve the detection and prognostic outcome of cervical cancer.

The kinesin superfamily proteins (KIFs) are a conserved class of ATP- and microtubule-dependent motor proteins that travel unidirectionally along microtubule tracks to fulfil their roles in intracellular transport or cell division [6]. To date, kinesins, comprise 45 members, have been categorized into 14 subfamilies (termed kinesin-1 to kinesin-14) by phylogenetic analysis of the motor domain [7]. KIFs are implicated in a variety of cellular functions, such as mitosis, signal transduction, microtubule polymer dynamics and intracellular transport, etc. [8]. Previous studies suggest that KIFs may play a key role in the development or progression in many kinds of cancer types [7,9,10]. Among them, KIF14 is localized at the central spindle and midbody along with its interaction with citron kinase (CIK) and protein-regulating cytokinesis 1 (PRC1) and was found to be involved in cytokinesis and chromosome segregation [11,12]. KIF14 has been found to be overexpressed due to genomic gain in multiple cancers, including breast, retinoblastoma, liver, renal, lung and ovarian cancers, etc. [13–18]. More recently, KIF14 has been reported to be involved in regulating chemoresistance by phosphorylating AKT in triple-negative breast cancer [19]. However, the expression of KIF14 and its clinical significance has not been investigated in cervical cancer.

In the present study, we assessed KIF14 expression in cervical cancer specimens and paired adjacent normal tissues as well as its expression in tissues samples from patients who are sensitive or resistant to paclitaxel treatment. We then examined the relationship between KIF14 expression, clinicopathological features or chemosensitivity and patient survival.

## METHODS

### Tissues specimens

The study was approved by the Ethical Committee of Jinan Second People's Hospital, and informed consent was obtained from all patients. Cervical cancer tissue samples and matched non-tumour adjacent tissues (NATs) were obtained from patients who underwent surgical resection at Jinan Second People's Hospital, between March 2010 and December 2014 and were diagnosed with cervical cancer based on histopathological evaluation. All tissues were immediately snap-frozen in liquid nitrogen and stored at −80°C until use. In addition, the patients with any other tumour were excluded from the study. A total of 47 pairs of cervical cancer tissues were examined in the study. According to the criteria of the Union for International Cancer Control (UICC)/American Joint Committee on Cancer (AJCC), seventh edition, 11, 9, 12 and 15 patients exhibited stage I, II, III and IV cancer respectively. None of the subjects had received any therapeutic procedures prior to the present study, including surgery, chemotherapy and radiotherapy. In addition, a total of 110 patients who had received paclitaxel treatment were enrolled informed consent was obtained before collecting samples. Patients with disease progression or recurrence 6 months or less after completing adjuvant chemotherapy were defined as being chemoresistant, whereas those without recurrence or recurrence more than 6 months after completing adjuvant chemotherapy were defined as chemosensitive. The prognosis was evaluated in all cervical cancer patients in June 2015. Overall survival (OS) was defined as the time from cancer onset until death or by censoring at the last follow-up date. The present study was blind to the designers.

### Total RNA extraction

Tissue sections were minced with scissors into small fragments (1–2 mm^3^) and homogenized with TRIzol™ reagent (Takara Bio). Chloroform (200 μl; Sigma–Aldrich) was added to the TRIzol homogenate. The preparations were then centrifuged at 12000×***g*** for 15 min at 4°C, and the upper aqueous layer was transferred to a clean Eppendorf tube, containing an equal volume of propan-2-ol (Sigma–Aldrich). The mixed suspensions were centrifuged at 12000×***g*** for a further 15 min at 4°C. The precipitations were then collected. After washing with 70% ethanol, total RNA was dissolved in RNase-free water and the quality of RNA was evaluated by gel electrophoresis. RNA concentrations were measured by optical density (260 nm, Q5000, Quawell) and the preparations stored at −80°C for subsequent analysis.

### RT-qPCR analysis

cDNA was reverse transcribed on the Bio-Rad S1000 Thermal Cycler (Bio-Rad Laboratories) using oligo (dT) as primers. Briefly, the total RNA (1 μg) from each sample was reverse transcribed in a 20 μl reaction volume, containing 0.5 μg of oligo (dT) and 200 U M-MLV (MBI Fermentas). All samples were amplified in triplicate under the following conditions: 95°C for 2 min, 35 cycles of 95°C for 15 s, 60°C for 30 s and 72°C for 20 s. qPCR reaction was performed on the Bio-Rad C1000 Real-Time Fluorescence Thermal Cycler (Bio-Rad Laboratories), using the following cycling conditions: initiation at 95°C for 10 min; amplification for 35 cycles, with denaturation at 95°C for 30 s; annealing at 56°C for 30 s; and elongation at 72°C for 30 s. A final extension at 72°C was performed for 10 min. GAPDH mRNA level was used for normalization.

### Immunoblot analysis

Whole tissue lysates were extracted with radioimmunoprecipitation assay (RIPA) buffer (0.1% sodium dodecyl sulfate, 0.5% deoxycholic acid, 1% Igepal, 150 mM NaCl and 50 mM Tris–HCl [pH 8]; Sigma), boiled and resolved on an 8–10% polyacrylamide gel, and transferred to polyvinylidene fluoride membrane. Antibodies against KIF14 (Abcam), phospho-Akt (Ser473, Cell Signaling), total-Akt (Cell Signaling) and GAPDH (Sigma–Aldrich) were used. The blots were incubated with horseradish peroxidase–conjugated donkey anti-rabbit or anti-mouse IgG (Santa Cruz Biotechnology) at a dilution of 1:5000 and detected with SuperSignalWest Pico Chemiluminescent Substrate Kit (Thermo Scientific).

### Statistical analysis

Statistical analysis was performed using IBM SPSS Statistics Version 16 (SPSS) and GraphPad Prism v5.0 (Graphpad Software). The Wilcoxon test was used to compare KIF14 expression in paired tumour tissue samples and NATs. The Mann–Whitney *U* test and Kruskal–Wallis test were used to perform statistical analysis of tissue KIF14 levels between unpaired groups and multiple comparison groups respectively. The Pearson's chi-squared test and Fisher's exact test were used to evaluate the association between tissue miRNA levels and clinicopathological parameters. In addition, survival curves were constructed with the Kaplan–Meier method and compared using log-rank test. Cox proportional hazards regression analysis was used for univariate and multivariate analyses of prognostic values. *P* value of two-sided less than 0.05 was considered statistically significant.

## RESULTS

### KIF14 expression in cervical cancer tissues and normal tissues

**Figure 1:**
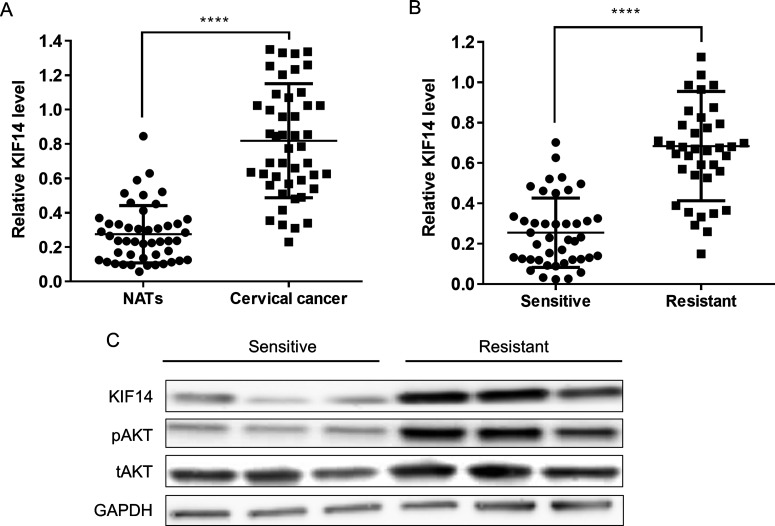
KIF14 expression levels in cervical cancer tissue samples and normal controls (**A**) Quantitative PCR analysis of relative KIF14 expression in tissue 47 pairs of cervical cancer patients and matched NATs. (**B**) Quantitative PCR analysis of relative KIF14 expression in tissue samples from cervical cancer patients who are sensitive (*n*=57) or resistant (*n*=53) to paclitaxel treatment. Data represent mean ± S.D. ****, *P*<0.0001. (**C**) Western blot analysis of KIF14, pAKT and total AKT protein expression in chemosensitive or chemoresistant patient samples. GAPDH was used as loading control.

We first evaluated the expression levels of KIF14 in 47 pairs of cervical cancer tissues and the NATs from patients who had not been exposed to any chemotherapy using quantitative real-time PCR. As shown in Figure 1(A), significantly higher levels of KIF14 were detected in tumour tissues compared with NATs (*P*<0.0001). KIF14 expression levels were also measured in tissue samples from a different population of participants consisting of 56 cervical cancer patients who are sensitive to paclitaxel treatment and 53 patients who are resistant to paclitaxel treatment. As shown in Figure 1(B), the expression levels of KIF14 were significantly higher in the cervical cancer patient group who are resistant to paclitaxel than the sensitive group (*P*<0.0001). Western blot analysis was also performed to confirm the differential expression level of KIF14 in a subset of these samples. As shown in Figure 1(C), there was an obvious up-regulation in the chemoresistant samples compared with chemosensitive ones. Correspondingly, an increase in activation of AKT was also observed in the chemoresistant group, consistent with previous report [19].

### Correlation between tissue KIF14 expression level and clinicopathological characteristics or chemosensitivity

The correlation of KIF14 expression levels with clinicopathological features or chemosensitivity of cervical cancer patients was summarized in Table 1. High levels of KIF14 were significantly correlated with TNM stage (*P*=0.0044), lymph node metastasis (*P*=0.0034) and chemoresistance (*P*<0.0001). However, no significant association was observed between KIF14 expression levels and other factors including age, tumour size and histology type (*P*=0.7588, 0.7657, 0.1476 respectively).

**Table 1:** Correlation between tissue KIF14 expression level and clinicopathological characteristics or chemosensitivity *, Statistical significance (*P*<0.05).

Characteristics	Number of patients	KIF14 low expression	KIF14 high expression	*P* value
Age (years)				
≤60	28	11	17	0.7588
>60	19	6	13	
Tumour size (cm)				
≤ 3	18	7	11	0.7657
>3	29	10	19	
Histology				
Adenocarcinoma	22	12	10	0.1476
Squamous carcinoma	25	8	17	
Tumour stage				
Stage I	11	9	2	0.0044*
Stage II, III and IV	36	11	25	
Lymph node metastasis				
Negative	23	17	6	0.0034*
Positive	24	7	17	
Chemosensitivity				
Sensitive	57	37	20	<0.0001*
Resistant	53	14	39	

### Prognostic significance of KIF14 expression in cervical cancer patients

**Figure 2:**
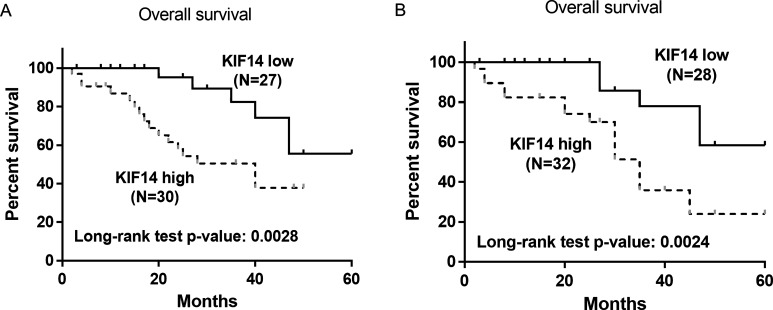
Kaplan–Meier analysis for survival based on KIF14 expression (**A**) Survival curves showing the correlation of KIF14 with OS in cervical cancer patients (*P*=0.0028). (**B**) OS based on KIF14 expression in cervical cancer patients treated with paclitaxel (*P*=0.0024).

Univariate and multivariate analyses were performed to determine the predictive factors for OS. The univariate analysis showed that TNM stage (*P*=0.025), lymph node metastasis (*P*=0.006) and KIF14 expression (*P*=0.012) were significantly associated with OS. Lymph node metastasis (*P*=0.003, HR=3.23, 95% confidence interval: 1.58–5.11) and KIF14 expression (*P*=0.009, HR=1.87, 95% confidence interval: 1.22–3.02) were independent factors as demonstrated by multivariate analysis. In addition, we found that the OS was significantly worse in patients with high levels of KIF14 (*P*=0.0028) with a median survival of 40 months. During the follow-up period, 62.2% of patients with high KIF14 had died whereas 44.4% of patients with low KIF14 had died (Figure 2A). These results indicated that high KIF14 expression was associated with poor OS and was an independent prognostic factor.

### Prognostic significance of KIF14 expression in cervical cancer patients treated with paclitaxel

Univariate and multivariate survival analyses were also performed in patients treated with paclitaxel. As shown in Table 2, TNM (*P*=0.011) and KIF14 expression (*P*=0.006) were significantly associated with OS. Multivariate analysis showed that KIF14 expression (*P*=0.002, HR=2.08, 95% confidence interval: 1.32–3.16) was an independent predictor of OS in paclitaxel treated patients (Table 2). Moreover, the estimated Kaplan–Meier OS curves indicated that high expression of KIF14 was significantly correlated with poor OS in patients treated with paclitaxel (*P*=0.0024) (Figure 2B). As of June 2015, 76.1% of patients with high KIF14 expression had died with a median survival of 35 months whereas 41.6% of patients with low KIF14 expression had died during the follow-up period (Figure 2B).

**Table 2:** Univariate and multivariate analysis of OS in cervical cancer patients *, Statistical significance (*P*<0.05). HR: hazard ratio.

Variables	HR	Univariate 95% CI	*P*	HR	Multivariate 95% CI	*P*
Age	1.22	0.68–1.59	0.61			
Tumour size	1.33	0.79–1.70	0.72			
Histology	1.16	0.63–1.49	0.58			
TNM stage	1.98	1.23–2.99	0.025*			0.825
Lymph node metastasis	3.99	2.25–6.98	0.006*	3.23	1.58–5.11	0.003*
KIF14 expression	2.03	1.34–4.22	0.012*	1.87	1.22–3.02	0.009*

## DISCUSSION

Over the past few years, accumulating evidence indicate that KIFs are involved in the initiation and progression of human cancers. For example, KIF3A and KIF3B have been reported to be implicated in oncogenesis and metastasis of breast cancer and renal carcinoma [20,21]. Overexpression of KIF11 has been shown to promote tumour development of multiple cancers [22]. KIF20A was found to be overexpressed in pancreatic ductal adenocarcinoma cells and its down-regulation inhibited the growth of gastric cancer cells [23,24]. In addition, some kinesin proteins are associated with malignancy development and drug resistance of solid tumour as well. De et al. [25] have shown that up-regulation of KIF3C could cause docetaxel resistance in breast cancer cells. Similar results were also previously reported on the association between taxane resistance in basal-like breast cancer and kinesins, including KIF3C, KIF5A and KIF12 [26].

KIF14, a member of the kinesin-3 family, contains a motor and a forkhead-associated domain and it plays an important role in cytokinesis as well as the segregation, congression and alignment of chromosomes [27–29]. Depletion of KIF14 has been shown to result in a delay in the metaphase-to-anaphase transition, inhibit cytokinesis and produce a binucleated phenotype [11,12]. Further mechanistic studies have shown that KIF14 may promote efficient cytokinesis through interactions with CIK and PRC1 [12]. KIF14 has been found to be up-regulated in various cancer types and is involved in tumorigenesis process partially due to its function in cytokinesis and chromosome segregation. Genomic gain of KIF14 and overexpression of this protein have been observed in breast, retinoblastoma, liver, renal, lung and ovarian cancers, etc. [13–18]. It has been reported that knockdown of KIF14 interferes with cell cycle progression and cytokinesis by blocking the p27 (Kip1) ubiquitination pathway in hepatocellular carcinoma [30]. Our results showed that significantly higher levels of KIF14 were detected in tumour tissues compared with NATs (Figure 1A). We also observed that high levels of KIF14 were significantly correlated with TNM stage and lymph node metastasis (Table 1). In addition, we found that the OS was significantly lower in patients with elevated levels of KIF14 (Figure 2A). Moreover, high KIF14 expression was associated with worse OS and was an independent prognostic factor by Cox proportional hazards risk analysis (Table 3). These results suggest that KIF14 may function as an oncogene and it may play important roles in cervical cancer progression and metastasis.

**Table 3:** Univariate and multivariate analysis of OS in cervical cancer patients who were treated with paclitaxel *, Statistical significance (*P*<0.05). HR: hazard ratio.

Variables	HR	Univariate 95% CI	*P*	HR	Multivariate 95% CI	*P*
Age	1.30	0.98-1.92	0.58			
Tumour size	1.78	1.14-2.88	0.32			
Histology	1.55	0.87-2.94	0.67			
TNM stage	2.06	1.34-3.05	0.011*			0.698
Lymph node metastasis	1.39	0.62-2.24	0.24			
KIF14 expression	2.39	1.47-3.69	0.006*	2.08	1.32-3.16	0.002*

Additional clinical investigations suggest that KIF14 can serve as prognostic biomarkers in various malignancies. For example, an earlier study led by Corson and Gallie [14] showed that KIF14 mRNA expression was a predictor of grade and outcome in breast cancer. Subsequent studies by the same group suggested that KIF14 messenger RNA expression was independently prognostic for outcome in lung cancer [15]. Moreover, KIF14 has been demonstrated as a candidate prognostic marker for outcome in glioma, ovarian cancer and hepatocyte carcinoma patients [31–33]. More recently, KIF14 has been identified as a regulator of doxetaxel chemosensitivity in triple-negative breast cancer [34]. Further studies indicated that up-regulation of KIF14 contributes to chemoresistance by promoting phosphorylation of AKT in triple-negative breast cancer [19]. In our study, we observed that both mRNA and protein levels of KIF14 were up-regulated in tissue samples from patients who are resistant to paclitaxel treatment compared with those who are sensitive (Figures 1B and 1C). Increased phosphorylation of AKT was also observed in the chemoresistant samples (Figure 1C). KIF14 expression levels were significantly associated with chemosensitivity in those patients (Table 1). In addition, high KIF14 expression levels predicted poor survival in patients with paclitaxel treatment (Figure 2B). Moreover, Cox proportional hazards risk analysis demonstrated that KIF14 was an independent prognostic factor for chemoresistance in cervical cancer (Table 2).

In conclusion, our study showed that KIF14 was up-regulated and was significantly associated with chemoresistance in cervical cancer. KIF14 may serve as a novel predictive factor for poor survival and a prognostic biomarker for chemoresistance in cervical cancer. Further studies are needed to understand the molecular mechanism underlying its role in cervical cancer development and chemoresistance and thus to support its potential clinical applications.

## Abbreviations

CIK: citron kinase
KIFs: kinesin superfamily proteins
NAT: non-tumour adjacent tissue
OS: overall survival
PRC1: protein-regulating cytokinesis 1
